# Altitude, Phenology, and Cotton Yield in Arid Oases: Quantifying Their Interactive Relationships

**DOI:** 10.3390/plants15050824

**Published:** 2026-03-07

**Authors:** Jian Huang, Pengfei Wu, Juan Huang, Wenyuan Xing, Hongfei Hao, Maochun Li, Xiaojun Wang

**Affiliations:** 1 Institute of Desert Meteorology, China Meteorological Administration, Urumqi 830002, China; wangxj@idm.cn; 2 Wulanwusu National Special Test Field for Comprehensive Meteorological Observation, Urumqi 830002, China; 3 Wulanwusu Ecology and Agrometeorology Observation and Research Station of Xinjiang, Urumqi 830002, China; 4 Urumqi Meteorological Satellite Ground Station, Urumqi 830011, China; wpf7848@sina.com (P.W.); xwy6851@sina.com (W.X.); 5 Xinjiang Climate Center, Urumqi 830002, China; at1321265319@163.com; 6 Bachu Meteorological Administration, Bachu 843800, China; haohongfei727@163.com; 7 Alar Meteorological Bureau, Alar 843300, China; lxm_xjale@sina.com

**Keywords:** cotton, phenological change, lint yield, altitude, arid oases area

## Abstract

Climate change induces cotton phenological changes, but the impact of these changes on yield and the regulatory role of altitude in the phenology–yield relationship remains unclear. Major Chinese cotton-growing regions (e.g., Xinjiang) are in arid and semi-arid areas with fragile ecosystems, making it crucial to clarify the phenology–yield correlation for ensuring regional cotton production security. Using long-term data (1981–2023) from 35 cotton monitoring stations in Xinjiang’s arid oases, we analyzed key phenological variations, quantified phenology’s impact on yield, and examined altitude’s effects on phenology. The results showed that the dates of four key cotton phenology—sowing (Sow), emergence (Eme), squaring (Squ), and flowering (Flo)—exhibited an advancing trend at a rate of 0.037–0.050 days year^−1^. In contrast, the dates of boll opening (Bol) and maturity (Mat) showed a delaying trend, with the delay rate ranging from 0.015 to 0.037 days year^−1^. Most phenological stage durations changed slightly: Sow–Eme, Squ–Flo, Bol–Mat, and vegetative growth period (VGP) shortened, while Eme–Squ, Flo–Bol, reproductive growth period (RGP), and whole growth period (WGP) lengthened. Lint yield increased by 24.061 kg ha^−1^ year^−1^. A one-day delay in the occurrence dates of any of the five cotton phenological stages—Sow, Eme, Squ, Flo, or Bol—was associated with a yield reduction ranging from 0.895 to 9.780 kg ha^−1^. In contrast, a one-day delay in the Mat led to a yield increase of 0.7876 kg ha^−1^. Additionally, the extension of three growth periods (Sow–Eme, Squ–Flo, and VGP) resulted in a yield decline, while the prolongation of four other periods (Eme–Squ, Bol–Mat, RGP, and WGP) contributed to a yield increase. The most critical finding is that altitude has a significant association with cotton phenology and its yield response: every 100 m increase in elevation, cotton phenological dates were delayed, the durations of different growth stages were altered, yield was reduced by 0.250 kg ha^−1^, and low-altitude areas exhibited more pronounced spatial heterogeneity in phenology and yield. However, this regulatory effect did not reach a significant level (*p* > 0.05), and the correlation between altitude and yield variability tended to be stronger in high-altitude areas than in low-altitude areas. This elevation-induced phenological shift is a key mediator of yield changes—elevational temperature variations are significantly associated with the duration of critical growth stages (e.g., the lengthening of reproductive growth period in low-altitude areas and shortening in high-altitude areas), which may indirectly affect dry matter accumulation and final yield formation. Corresponding policies for different altitudes should be formulated to offset the negative effects of phenological changes, providing scientific support for securing cotton production in arid oases.

## 1. Introduction

Amid global climate change, agricultural production stability and sustainability face mounting challenges, making crop yield responses to environmental changes a key focus in agricultural ecology and climate change research [1]. As an intuitive indicator of crop growth, phenology regulates yield formation by determining the spatiotemporal distribution of vegetative and reproductive growth, and thus influencing resource (light, heat, water) use efficiency [2]. For cotton (*Gossypium hirsutum* L.), a globally vital cash crop and natural fiber source, yield formation depends on an orderly sequence of phenological stages (sowing, emergence, squaring, flowering, boll opening, and maturity). Shifts in the timing or duration of these key stages can significantly alter lint yield by modifying resource allocation and affecting reproductive organ development (e.g., pollen viability, boll-setting rate).

Existing studies confirm that spatiotemporal phenological variations are key intrinsic drivers of crop yield fluctuations. Notably, cotton’s flowering and boll-forming stage is pivotal for yield formation, with its duration and environmental adaptability directly governing boll number and weight. [3]. Driven by climate change, rising temperatures have further intensified cotton phenological variability. For example, during the pollen germination stage, 35 °C may be the critical threshold for pollen viability transition [4]. However, significant research gaps remain. On the one hand, studies on the quantitative relationship between phenological phases (individual phases and stage durations) and cotton yield are fragmented, lacking systematic coupling analysis. On the other hand, there is insufficient exploration of how the phenology–yield relationship varies across different geographical contexts (e.g., altitude gradients, arid oasis regions), making it difficult to identify region-specific phenological regulation targets and thus limiting the formulation of precise cultivation management strategies.

Climate conditions vary with altitude, thus affecting crop phenology. Spring wheat exhibits distinct responses to climate warming across altitude gradients. In low-altitude regions, when sowing is advanced, maturity is nearly unchanged, the growth period is prolonged, and yield declines. In contrast, high-altitude regions see delayed sowing, advanced maturity, shortened growth periods, and significant yield fluctuations. Notably, a 1 °C rise in the growing-season daily mean temperature reduces the growth period of high-altitude spring wheat by 11.7 days, with no such change detected in low-altitude areas [5]. Altitude affects the key phenological stages of loquat (flowering and fruit expansion stages) by altering the temperature environment. In high-altitude areas, low-temperature risk during flowering intensifies freezing injury, whereas the absence of high-temperature stress during fruit expansion mitigates heat damage. Notably, in specific temperature inversion zones (150–250 m), air temperature increases with rising altitude at a rate of 0.5–1.5 °C per 100 m. This unique temperature profile significantly reduces freezing injury, leading to a 3–5-day advancement in phenological stages (e.g., ripening) relative to non-inversion zones at the same altitude [6]. For every 100 m increase in altitude, maize phenological stages are delayed by 4–5 days [7]. Altitude determines the spatial distribution of key climatic indicators for cotton growing periods: for every 100 m increase in altitude, accumulated temperature ≥ 10 °C decreases by approximately 200 °C day^−1^, and the frost-free period shortens by 10–15 days [8]. High-altitude environments prolong plant growth cycles, reduce growing degree-day requirements, further promote dry matter accumulation in ears and overall vegetative growth, and enhance heat use efficiency [9]. Asse et al. [10] argued that global warming weakens the altitudinal gradient of spring phenology. Qiu et al. [11] clarified the differences in crop planting patterns along different altitudinal gradients. Zheng et al. [12] revealed the regulatory pathway of altitude on crop flowering phenology at the molecular level. Nevertheless, few studies have specifically addressed the associations between cotton phenological traits and altitude gradients.

China is the world’s largest cotton producer and consumer, accounting for approximately 25% and 30% of the global cotton output and consumption, respectively [13,14]. Specifically, Xinjiang—one of its major cotton-growing regions—lies in arid and semi-arid zones, with scarce precipitation and prominent soil salinization leading to significant ecological vulnerability [15]. Cotton phenology here is highly susceptible to climate change: rising temperatures may dislocate growth stages, and summer heat can inhibit boll development, exacerbating yield fluctuation risks [16,17].

Therefore, systematically analyzing the long-term variation characteristics of key phenological stages of cotton in arid oasis regions, quantifying the contribution weight of different phenological factors to yield changes, and identifying yield-dominant key phenological stages are essential steps. In addition, clarifying how geographical factors (e.g., altitude) regulate the phenology–yield relationship mechanism carries great theoretical and practical significance. It can support the optimization of regional cotton cultivation management (e.g., targeted regulation of key phenological stages, breeding of adaptive varieties), help improve yield stability, and ensure cotton production security. Based on this, this study takes cotton in arid oasis regions as the research object, focuses on the core of the phenology–yield relationship, explores the impact mechanisms of key phenological stage changes on yield, and provides scientific support for constructing a high-yield and stable-yield cotton cultivation system adapted to climate change.

Existing studies have widely focused on the impacts of climate change on cotton phenology, yielding certain consensus and region-specific conclusions. For example, Huang and Ji [18] studied Wulanwusu Station in the arid oasis of Xinjiang and found that all cotton phenological stages advanced. The sowing-emergence and boll opening-boll filling stages shortened by 1.76 days and 5.19 days, respectively, while other growth stages prolonged by 2–9.71 days, extending the total growth period by 22.26 days overall. In contrast, Ahmad et al. [19] analyzed thermal trend effects on cotton phenology in central and southern Punjab, Pakistan. They concluded that all key phenological stages advanced, and each growth phase shortened in duration. Li et al. [20] further noted that cotton emergence, squaring, flowering, and boll opening stages advanced, whereas sowing and maturity stages were delayed. The sowing-emergence and squaring-flowering stages were shortened, while other growth stages were prolonged. They also pointed out that seed cotton yield decreased with delayed sowing, emergence, squaring, flowering, and boll opening stages, but increased with extended durations of sowing-emergence, flowering-boll opening, boll opening-maturity, and sowing-maturity stages. Generally, climate warming accelerates cotton growth, advances phenological stages, and shortens the total growth period, with such changes showing significant regional heterogeneity [21]. Moreover, targeted research is lacking on core issues in Xinjiang’s arid oases, such as how altitude regulates the phenology–yield relationship and whether there are altitude-specific phenological stages dominating yield formation.

Therefore, this study aimed to achieve the following four objectives: (a) to determine the long-term variation trends of cotton phenology and lint yield; (b) to quantify the impacts of phenological changes on cotton lint yield; (c) to identify the phenological phase with the highest contribution rate to the cotton lint yield variation; d) to reveal the regulatory effect of altitude, a key geographical factor, on the long-term variation trends of cotton phenology and lint yield.

## 2. Materials and Methods

### 2.1. Cotton Phenology, Cotton Yield, and Climate Data

Phenological data for cotton (35 stations) of Xinjiang, China, spanning 1981–2023, were retrieved from the China Meteorological Administration website (http://data.cma.cn, accessed on 1 January 2026) following strict quality control and inspection procedures. Phenological data comprised dates of six key phenological events, namely sowing (Sow), emergence (Eme), squaring (Squ), flowering (Flo), boll opening (Bol), and maturity (Mat). Based on these data, several important phenological phases were delineated, including Sow–Eme (sowing to emergence), Eme–Squ (emergence to squaring), Squ–Flo (squaring to flowering), Flo–Bol (flowering to boll opening), and Bol–Mat (boll opening to maturity), the vegetative growing period (VGP, sowing to flowering), the reproductive growing period (RGP, flowering to maturity), and the whole growing period (WGP, sowing to maturity).

**Figure 1:**
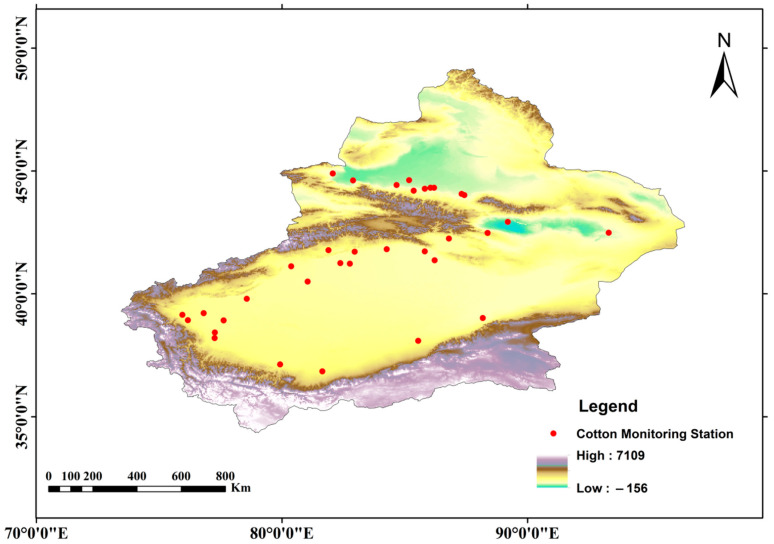
Monitoring site distribution of cotton in oases of arid areas, Xinjiang, China.

Due to the unique terrain of “three mountains surrounding two basins”, Xinjiang exhibits two different climatic regimes in southern and northern Xinjiang (Figure 1). For the 35 study stations, detailed geographical characteristics, long-term mean climatic factors over the entire cotton growing season, and key phenological events were summarized in Table 1.

### 2.2. Methods

#### Temporal Trends in Cotton Phenology and Yield

The average dates for Sow, Eme, Squ, Flo, Bol, and Mat of cotton were determined for each station. Using the delineated phenological growth stages for the eight growth stages (Sow–Eme, Eme–Squ, Squ–Flo, Flo–Bol, Bol–Mat, VGP, RGP, and WGP) were calculated for each station. Cotton plants were observed every other day during the entire growing season each year, and the calendar dates were recorded when 50% of the plants in the observation area had changed developmental stages. Each growth stage of cotton was recorded following descriptions by Liu et al. [22].

In this study, linear regression analysis was employed to examine the variation trends of different phenological stages of cotton at 35 stations, as well as the variation trends of cotton lint yield.

Pearson correlation analysis, regression models, and Partial Least Squares (PLS) regression were used to examine phenology–yield relationships. PLS regression additionally served to mitigate multicollinearity and determine the relative contribution of each phenological factor. In PLS analysis, the maximum number of latent factors was set to 14 (corresponding to 14 phenological stages, and the number of latent variables was determined by cross-validation with 10 folds). All other parameters were calculated using the default settings in SPSS 26.0 (SPSS Inc., Chicago, IL, USA). All statistical analyses were conducted with SPSS 26.0 for Windows. To eliminate the interference caused by differences in dimensions and numerical ranges, all data were standardized using the Z-score method. PLS facilitates comparative analysis of multiple response and explanatory variables [23], demonstrates strong resistance to overfitting, and outperforms principal component analysis (PCA) in performance [24]. Statistical significance was defined as *p* < 0.05, and all graphs were generated using SigmaPlot 12.5 for Windows (Systat Software, Inc., San Jose, CA, USA).

Specifically, we have expanded the *K*-means clustering analysis method description as follows: First, for the *K*-means clustering analysis of altitude data from 35 cotton monitoring stations, we first preprocessed the altitude data to exclude outliers (using the 3σ criterion) to avoid interference with clustering results. We then tested potential *k* values in the range of 2 to 10, and combined two methods to determine the optimal *k*: ① the elbow method, which evaluates clustering effectiveness by calculating the sum of squared errors (SSE) between each sample and its cluster center—we identified the *k* value where the SSE curve showed a clear inflection point (i.e., the “elbow”) as a candidate; ② the silhouette coefficient method, which quantifies the compactness within clusters and separation between clusters, with a higher silhouette coefficient indicating better clustering quality. Through comprehensive comparison, the optimal *k* value was determined to be 2, as this *k* achieved both a significant SSE decrease and the highest silhouette coefficient (0.72, indicating a good clustering effect).

Second, regarding the calculation of the altitude division threshold: after executing *K*-means clustering with *k* = 2, we extracted the maximum altitude of the low-altitude cluster (852.34 m) and the minimum altitude of the high-altitude cluster (881.29 m), and calculated their arithmetic mean (866.817 m) as the final altitude division threshold. This threshold was selected to minimize the overlap between the two altitude groups and ensure clear differentiation.

Third, for the verification of phenological slope differences between groups, we first calculated the phenological slope (i.e., the interannual change rate of each phenological stage) for each station using linear regression analysis. We then performed an independent samples *t*-test to compare the phenological slopes between the two altitude groups—prior to the test, we verified the normality (Shapiro–Wilk test, *p* > 0.05) and homogeneity of variance (Levene’s test, *p* > 0.05) of the phenological slope data, confirming that the data met the assumptions of the *t*-test. The results showed that the phenological slopes of most key stages differed significantly between the two groups (*p* < 0.05), validating the rationality of the altitude division.

## 3. Results

### 3.1. Trends in Changes of Cotton Phenology and Cotton Lint Yield

**Figure 2:**
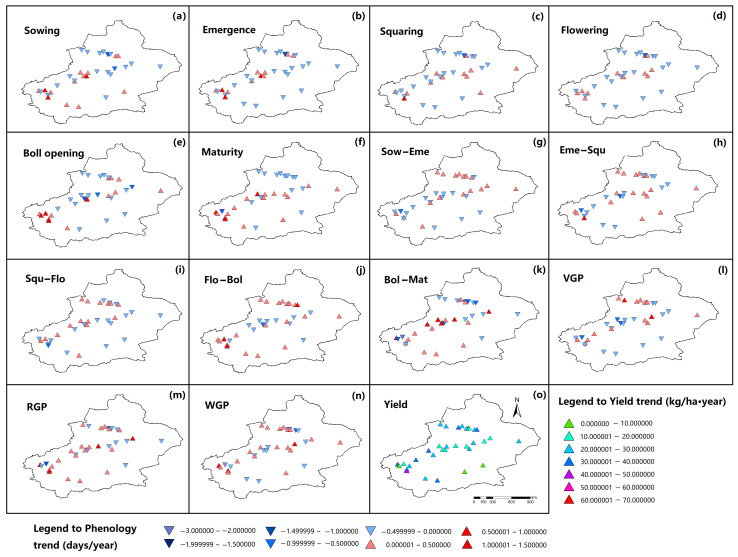
The variations in cotton phenology and yield across all sites. (**a**) Variation trends in sowing dates; (**b**) variation trends in emergence dates; (**c**) variation trends in squaring dates; (**d**) variation trends in flowering dates; (**e**) variation trends in boll opening dates; (**f**) variation trends in maturity dates; (**g**) variation trends in duration of Sow−Eme phase; (**h**) variation trends in duration of Eme−Squ phase; (**i**) variation trends in duration of Squ−Flo phase; (**j**) variation trends in duration of Flo−Bol phase; (**k**) variation trends in duration of Bol−Mat phase; (**l**) variation trends in duration of VGP phase; (**m**) variation trends in duration of RGP phase; (**n**) variation trends in duration of WGP phase; (**o**) variation trends in cotton lint yield. Downward-pointing triangles indicate advanced or shortened phenological phases, while upward-pointing triangles represent delayed or prolonged phenological phases. Color intensity reflects the magnitude of change.

**Figure 3:**
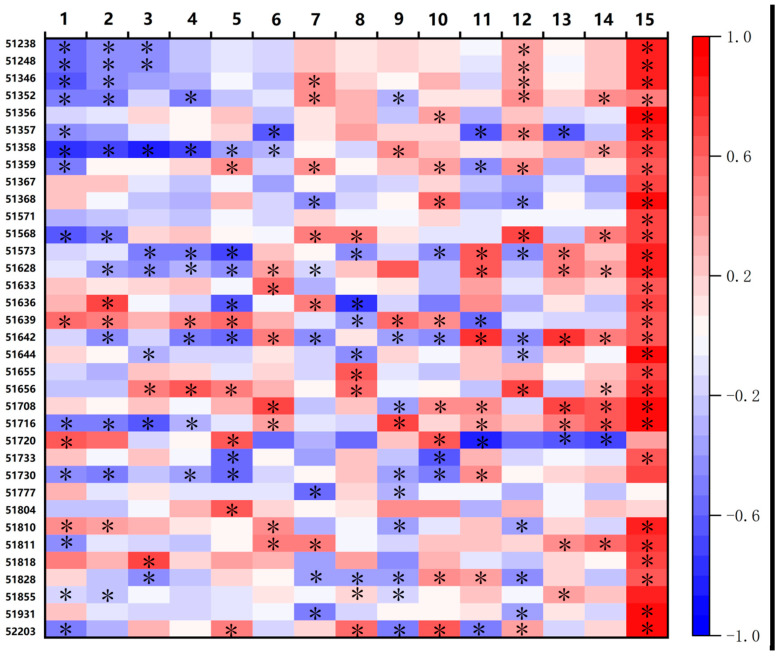
Correlation coefficients and significance levels between phenology and year (Columns 1–14), as well as between yield and year (Column 15) at each site. 1: Sowing; 2: Emergence; 3: Squaring; 4: Flowering; 5: Boll opening; 6: Maturity; 7: Sow–Eme; 8: Eme–Squ; 9: Squ–Flo; 10: Flo–Bol; 11: Bol–Mat; 12: VGP; 13: RGP; 14: WGP; 15: Yield. * indicates *p* < 0.05. Color intensity indicates the strength of the correlation, with blue for negative correlation and red for positive correlation. The scale beside it represents the correlation ranging from −1 to 1.

The variations in cotton phenology and yield trend across all sites were illustrated in Figure 2. The Sow, Eme, Squ, and Flo dates advanced by an average of 0.037, 0.045, 0.034, and 0.050 days year^−1^, respectively; whereas Bol and Mat dates delayed by an average of 0.037 and 0.015 days year^−1^, respectively (Table 2). The mean phenology date at each site is in Table S1. Across the 35 sites, Sow, Eme, Squ, Flo, Bol, and Mat advanced at 21, 24, 22, 22, 19, and 18 sites, respectively, whereas the number of sites with delayed dates for these phenological dates was 14, 11, 13, 13, 16, and 17, respectively (Table 2). Specifically, significant advances in Sow, Eme, Squ, Flo, Bol, and Mat dates were detected at 12, 11, 8, 7, 7, and 2 sites, respectively, compared with 3, 2, 2, 2, 6, and 7 sites with significant delays in these phenological events, respectively (Table 2), and the advancement or delay of phenology dates and its significance level at each site were shown in Figure 3. The durations of Sow–Eme, Squ–Flo, Bol–Mat, and VGP shortened by an average of 0.002, 0.029, 0.015, and 0.004 days year^−1^, respectively, while those of Eme–Squ, Flo–Bol, RGP, and WGP lengthened by an average of 0.012, 0.084, 0.060, and 0.035 days year^−1^, respectively (Table 2). Among the 35 sites, the phenological phases of Sow–Eme, Eme–Squ, Squ–Flo, Flo–Bol, Bol–Mat, VGP, RGP, and WGP were lengthened at 18, 20, 16, 23, 18, 16, 21, and 20 sites, respectively, whereas the number of sites where these phases were shortened was 17, 15, 19, 12, 17, 19, and 14, respectively (Table 2). For the above eight phenological phases, the number of sites with a significant lengthening was 6, 5, 3, 8, 7, 9, 7, and 8, respectively, while the number of sites with a significant shortening was 6, 5, 9, 4, 5, 5, 7, and 1, respectively (Table 2), and the shortening or prolongation of each phenological stage and the corresponding significance levels for all 35 cotton observation sites were presented in Figure 3. These results suggested that the cotton phenological progression displayed stage-dependent variations, with considerable spatial heterogeneity among the 35 monitoring sites. Figure 3 illustrates the correlation coefficients and their corresponding significance levels for the relationships between phenological stages and year, and between yield and year across all sites.

The yield change trend across sites ranged from 0.118 to 61.094 kg ha^−1^ year^−1^, with a mean value of 24.061 kg ha^−1^ year^−1^ and a standard deviation of 11.481 kg ha^−1^ year^−1^. All 35 sites exhibited an increasing trend in cotton lint yield, with substantial variability in the magnitude of this trend across sites (Figure 2), and 30 of these sites showed a statistically significant increasing trend (Figure 3).

### 3.2. Impacts of Delays or Prolongation in Phenology on Cotton Lint Yield

**Figure 4:**
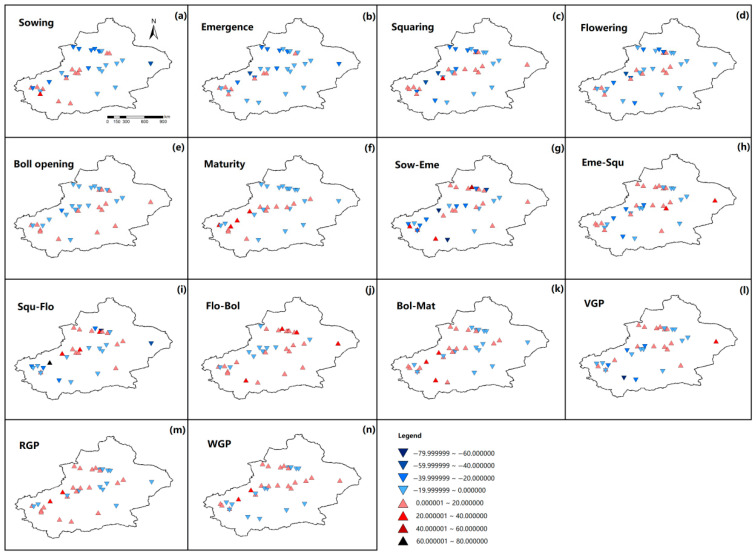
Impacts of delays or prolongation in phenology on cotton lint yield. (**a**) Effect of sowing date delay on cotton lint yield; (**b**) effect of emergence date delay on cotton lint yield; (**c**) effect of squaring date delay on cotton lint yield; (**d**) effect of flowering date delay on cotton lint yield; (**e**) effect of boll opening date delay on cotton lint yield; (**f**) effect of maturity date delay on cotton lint yield; (**g**) effect of Sow–Eme phase prolongation on cotton lint yield; (**h**) effect of Eme–Squ phase prolongation on cotton lint yield; (**i**) effect of Squ–Flo phase prolongation on cotton lint yield; (**j**) effect of Flo–Bol phase prolongation on cotton lint yield; (**k**) effect of Bol–Mat phase prolongation on cotton lint yield; (**l**) effect of VGP phase prolongation on cotton lint yield; (**m**) effect of RGP phase prolongation on cotton lint yield; (**n**) effect of WGP phase prolongation on cotton lint yield. Downward-pointing triangles indicate a decrease in yield, while upward-pointing triangles represent an increase in yield. Color intensity reflects the magnitude of change.

**Figure 5:**
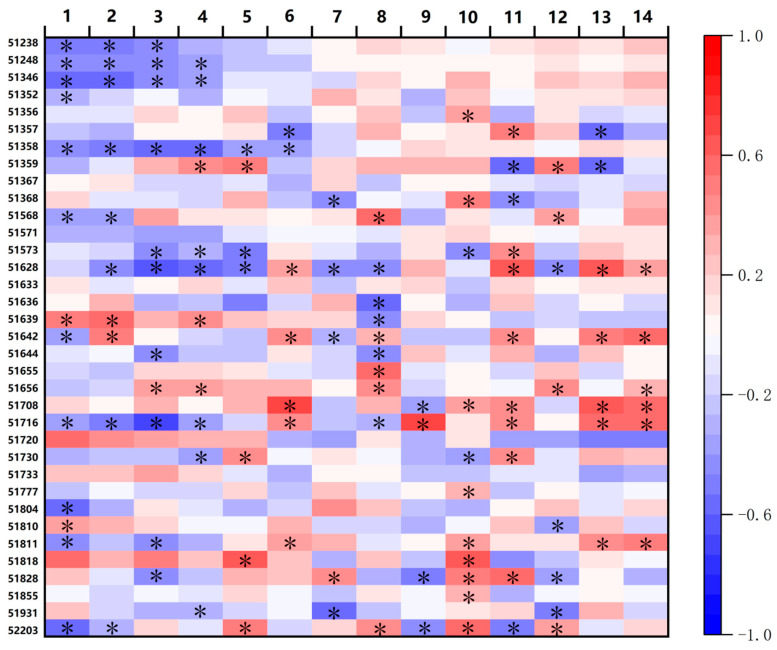
Correlation coefficients and significance of the effects of phenological delays or lengthening on cotton lint yield. * indicates *p* < 0.05. 1: Sowing; 2: Emergence; 3: Squaring; 4: Flowering; 5: Boll opening; 6: Maturity; 7: Sow–Eme; 8: Eme–Squ; 9: Squ–Flo; 10: Flo–Bol; 11: Bol–Mat; 12: VGP; 13: RGP; 14: WGP; 15: Yield. * indicates *p* < 0.05. Color intensity indicates the strength of the correlation, with blue for negative correlation and red for positive correlation. The scale beside it represents the correlation ranging from −1 to 1.

For each one-day delay in Sow, Eme, Squ, Flo, and Bol dates, cotton lint yield decreased by −7.620, −9.780, −5.502, −7.441, and −0.895 kg ha^−1^, respectively; in contrast, each one-day delay in Mat date increased lint yield by 0.7876 kg ha^−1^. The number of sites where yield increased with the delay in Sow, Eme, Squ, Flo, Bol, and Mat dates was 12, 9, 16, 12, 17, and 14, respectively, among which the number of sites with a significant increase was 2, 2, 1, 2, 2, 4, and 5, respectively. In contrast, the number of sites where yield decreased with the delay in these phenological dates was 23, 26, 19, 23, 18, and 21, respectively, with the number of sites showing a significant decrease being 11, 8, 10, 8, 3, and 2 (Table 3). For each one-day extension of the Sow–Eme, Squ–Flo, and VGP stages, cotton lint yield decreased by −5.724, −4.422, and −3.067 kg ha^−1^, respectively; in contrast, each one-day extension of the Eme–Squ, Bol–Mat, RGP, and WGP stages increased lint yield by 0.744, 5.769, 2.010, 3.392, and 3.165 kg ha^−1^, respectively (Table 3). The number of sites where yield increased with the prolongation of the Sow–Eme, Eme–Squ, Squ–Flo, Flo–Bol, Bol–Mat, VGP, RGP, and WGP stages was 17, 19, 17, 24, 20, 17, 21, and 21, respectively, among which the number of sites with a significant increase reached 1, 5, 1, 9, 8, 4, 5, and 6, respectively. In contrast, the number of sites where yield decreased with the prolongation of the above-mentioned stages was 19, 16, 18, 11, 15, 18, 14, and 14, respectively, with the number of sites showing a significant decrease being 4, 4, 3, 2, 3, 4, 2, and 0, respectively (Table 3). The maximum and minimum values presented in Table 3 demonstrated substantial differences among sites in terms of the impact of phenological variations on cotton lint yield. These results were also supported by Figure 4. The yield responses (increase or decrease) and their significance levels associated with phenological delay or stage prolongation at each individual site, as further confirmed by the correlation coefficients for the impacts of phenological delays or lengthening on cotton lint yield, were illustrated in Figure 5. The spatial heterogeneity of phenological changes (variations in phenological trends across different sites) directly lead to spatial variability in yield changes: most sites showed an increasing yield trend due to “advancements in early phenological stages + delays in late phenological stages + lengthening of reproductive growth periods”, while a small number of sites exhibited a decreasing yield trend as a result of “delays in early phenological stages + delays in boll opening stage”.

**Figure 6:**
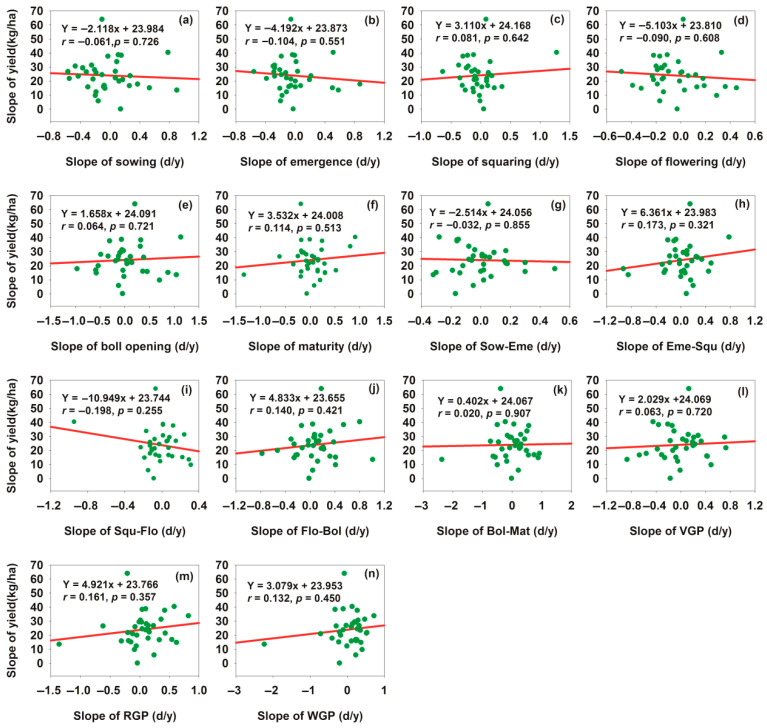
Relationship between the slopes of phenological changes and cotton lint yield. (**a**) Relationship between sowing date slope and yield slope; (**b**) relationship between emergence date slope and yield slope; (**c**) relationship between squaring date slope and yield slope; (**d**) relationship between flowering date slope and yield slope; (**e**) relationship between boll opening date slope and yield slope; (**f**) relationship between maturity date slope and yield slope; (**g**) relationship between Sow–Eme duration slope and yield slope; (**h**) relationship between Eme–Squ duration slope and yield slope; (**i**) relationship between Squ–Flo duration slope and yield slope; (**j**) relationship between Flo–Bol duration slope and yield slope; (**k**) relationship between Bol–Mat duration slope and yield slope; (**l**) relationship between VGP duration slope and yield slope; (**m**) relationship between RGP duration slope and yield slope; (**n**) relationship between WGP duration slope and yield slope.

To quantify the driving relationship between long-term phenological change trends and long-term yield trends, and to clarify the direction, strength, and spatial heterogeneity of this relationship, linear correlation analysis was performed between phenological slopes and yield slopes. For the 35 sites, increases in the slopes of delay of Sow, Eme, and Flo dates and the slopes of lengthening of Sow–Eme and Squ–Flo stages reduced cotton lint yield, whereas increases in the slopes of delay of Squ, Bol, and Mat dates and the slopes of lengthening of Eme–Squ, Flo–Bol, Bol–Mat, VGP, RGP, and WGP stages increased cotton lint yield (Figure 6). The greatest yield reduction was observed in the Squ–Flo stage, with a decrease of 10.949 kg ha^−1^ year^−1^ per one-day slope lengthening (Figure 6i); while the largest yield increase occurred in the Eme–Squ stage, with an increase of 6.361 kg ha^−1^ year^−1^ per one-day slope lengthening (Figure 6h). Therefore, the Squ–Flo and Sem-Squ stages were the most sensitive phenological stages for yield reduction and yield increase, respectively. These results demonstrated a synchronous relationship between long-term phenological changes and cotton lint yield trends: delays in Sow, Eme, and Flo dates and lengthening of Sow–Eme and Squ–Flo stages were associated with a decreasing trend in cotton lint yield, whereas delays in Squ, Bol, and Mat dates and lengthening of Eme–Squ, Flo–Bol, Bol–Mat, VGP, RGP, and WGP stages were associated with an increasing trend in cotton lint yield. Therefore, the correlation between the two types of slopes in Figure 6 not only reflected the directionality of their variation trends but also indicated that each phenological stage exerted a distinct impact on cotton lint yield.

### 3.3. Effects of Elevation on Cotton Phenology and Yield

**Figure 7:**
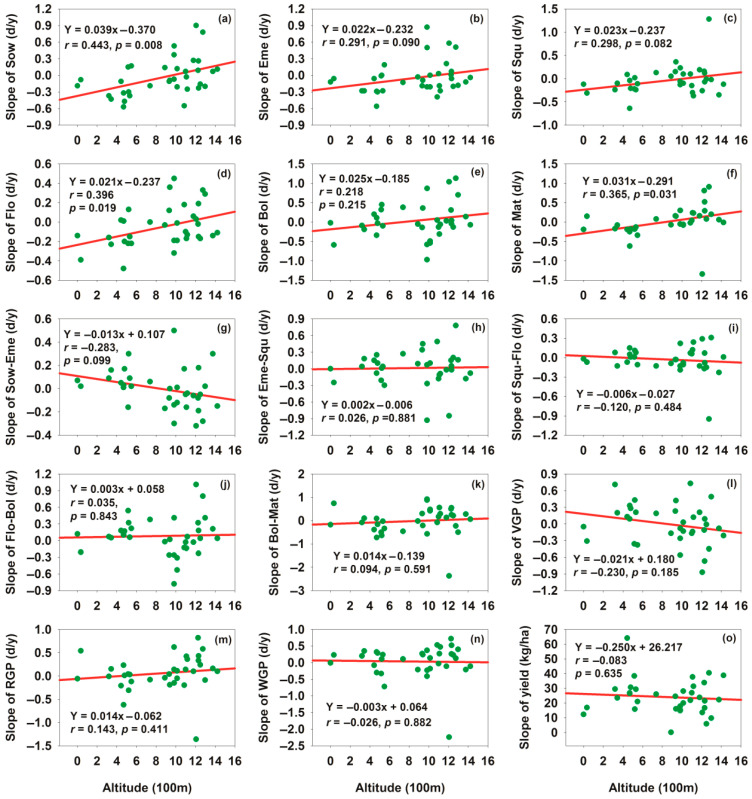
Relationships between elevation and phenological slopes as well as cotton lint yield slopes. (**a**) Relationship between sowing date slope and altitude; (**b**) relationship between emergence date slope and altitude; (**c**) relationship between squaring date slope and altitude; (**d**) relationship between flowering date slope and altitude; (**e**) relationship between boll opening date slope and altitude; (**f**) relationship between maturity date slope and altitude; (**g**) relationship between Sow–Eme duration slope and altitude; (**h**) relationship between Eme–Squ duration slope and altitude; (**i**) relationship between Squ–Flo duration slope and altitude; (**j**) relationship between Flo–Bol duration slope and altitude; (**k**) relationship between Bol–Mat duration slope and altitude; (**l**) relationship between VGP duration slope and altitude; (**m**) relationship between RGP duration slope and altitude; (**n**) relationship between WGP duration slope and altitude; (**o**) relationship between yield slope and altitude.

Linear correlation analysis was conducted between altitude and the slopes of cotton phenology and lint yield to reveal the regulatory effect of altitude, a key geographical factor, on the long-term change trends of cotton phenology and lint yield, and further to explain the causes of spatial heterogeneity in the phenology–yield driving relationship. Overall, for every 100 m increase in elevation, the Sow, Eme, Squ, Flo, Bol, and Mat dates were delayed by 0.039, 0.022, 0.023, 0.021, 0.025, and 0.031 days year^−1^, respectively; the Eme–Squ, Flo–Bol, Bol–Mat, and RGP were prolonged by 0.002, 0.003, and 0.014 days year^−1^, respectively; while the Sow–Eme, Squ–Flo, VGP, and WGP were shortened by 0.013, 0.006, 0.021, and 0.003 days year^−1^, respectively. Concurrently, the lint yield decreased by 0.250 kg ha^−1^ per 100 m elevation increase; additionally, the Sow, Flo, and Mat dates showed a significant positive correlation with increasing elevation (Figure 7a,d,f). For Figure 7a,d,f, the residual normality test (Shapiro–Wilk test), homoscedasticity test (Pearson correlation), and serial autocorrelation test (Durbin–Watson test) were conducted, all of which satisfied the classical assumptions of simple linear regression, confirming the reliability of the significant regression results and the absence of false significance risk. These findings demonstrated that variations in elevation had a significant association with both cotton phenological development and lint yield. Elevation affected yield primarily through two pathways: (1) Directly altering the thermal environment, which changed the duration of yield-dominant phenological stages (e.g., reproductive growth period); (2) Inducing spatial heterogeneity in phenology–yield relationships (more significant in low-altitude areas), which amplified the impact of phenological shifts on yield stability. The 0.250 kg ha^−1^ yield reduction per 100 m elevation increase was mainly attributed to the shortening of reproductive growth period caused by lower accumulated temperature at higher altitudes, which limited boll development and lint accumulation.

*K*-means clustering analysis was performed on the altitude data of 35 cotton stations to determine the optimal number of groups (*k*) and the corresponding altitude division thresholds. The optimal *k* value was identified by combining the elbow method (based on the sum of squared errors, SSE) and the silhouette coefficient method. After determining the optimal *k* (*k* = 2 in this study), *K*-means clustering was executed, and the altitude division threshold was calculated as the average of the maximum altitude of the low-altitude group and the minimum altitude of the high-altitude group. A *t*-test was used to verify the significant differences in phenological slopes between different altitude groups. The results showed that the altitude of the 35 stations could be divided into two intervals: <866.817 m and ≥866.817 m.

**Figure 8:**
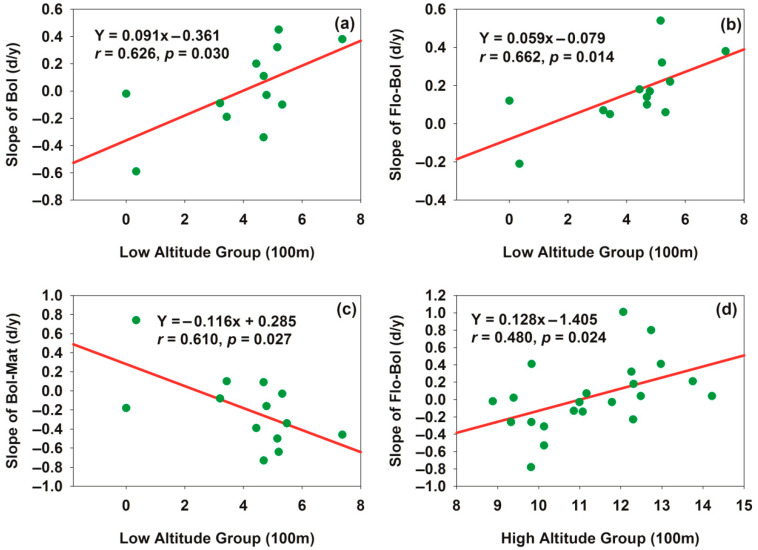
Phenology showing different responses to high-altitude group and low-altitude group. (**a**) Relationship between low altitude and boll date slope; (**b**) relationship between low altitude and Flo–Bol duration slope; (**c**) relationship between low altitude and Bol–Mat duration slope; (**d**) relationship between high altitude and Flo–Bol duration slope.

To reveal the spatial heterogeneity of cotton phenology and its response to altitude, 35 cotton stations were divided into a low-altitude group (cluster center: 416.2 m, 13 stations) and a high-altitude group (cluster center: 1133.09 m, 22 stations) based on *K*-means clustering analysis. Linear regression analysis showed that for every 100 m increase in altitude, in the low-altitude group, the slope of the Bol date increased significantly by 0.091 (i.e., a delay of 0.091 days) (Figure 8a), the slope of the Fol-Bol duration increased significantly by 0.059 (i.e., a prolongation of 0.059 days) (Figure 8b), and the slope of the Bol–Mat duration decreased significantly by 0.116 (i.e., a shortening of 0.116 days) (Figure 8c). In contrast, in the high-altitude group, only the slope of the Fol-Bol duration increased significantly by 0.128 (i.e., a prolongation of 0.128 days) (Figure 8d). For Figure 8a–d, the residual normality test (Shapiro–Wilk test), homoscedasticity test (Pearson correlation), and serial autocorrelation test (Durbin–Watson test) were conducted, all of which met the classical assumptions of simple linear regression, verifying the reliability of the significant regression results with no risk of false significance. These results indicated that the regulatory effect of altitude on cotton phenology had obvious spatial heterogeneity: in the plain oasis core cotton area (low-altitude group), altitude affected cotton phenology throughout the whole growth period, while in the mountain edge oasis cotton area (high-altitude group), altitude only affected the key transition period from squaring to flowering. Notably, the stronger response of Fol-Bol duration to altitude in the high-altitude group (slope = 0.128) (Figure 8d) compared to the low-altitude group (slope = 0.059) (Figure 8b) suggests that this specific phenological phase may be more sensitive to environmental changes in high-altitude areas. The differentially significant responses of cotton phenology to altitude between the low- and high-altitude groups clearly indicated the existence of spatial heterogeneity in cotton phenology across the altitude gradient in the study area.

### 3.4. Contribution Rates of Phenological Phases to Cotton Lint Yield

**Figure 9:**
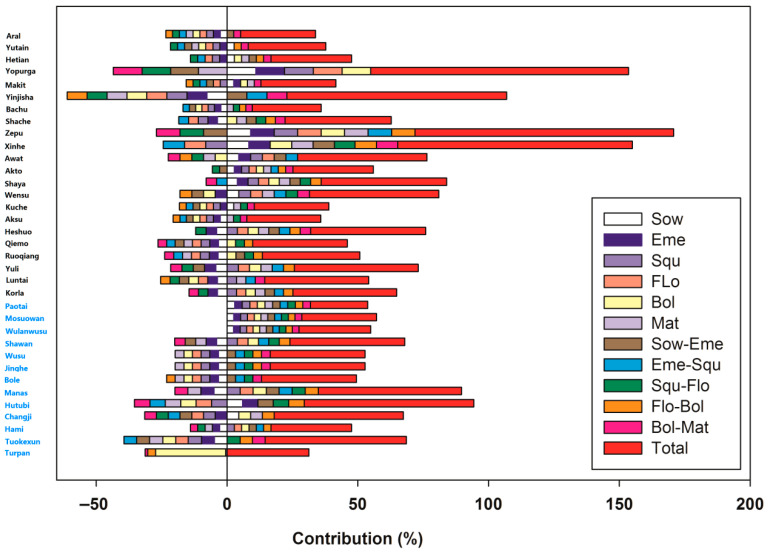
Contribution rates of phenological phases to cotton lint yield. The positive and negative values of the contribution rate indicate a consistent or opposite direction to the yield increase trend. The sum of the absolute values of the contribution rates of phenological phases to yield at each site represents the total contribution rate of phenology at that site. Station names in black denote the high-altitude group, while those in blue represent the low-altitude group.

The contribution rates of cotton phenological phases to yield across the 35 sites were illustrated in Figure 9. All phenological indicators (X) and yield data (Y) were standardized using Z-score normalization before PLS analysis to eliminate the effects of dimensional differences and magnitude variations.

Spatial heterogeneity of total contribution 

**Figure 10:**
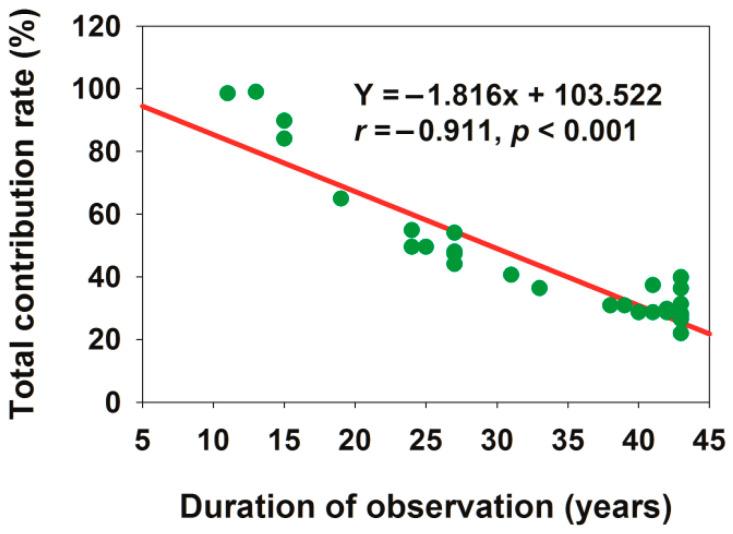
Relationship between observation duration and total contribution rate.

The total contribution rates varied significantly among different sites: the total contribution rates of Zepu, Yopurga, Xinhe, and Yinjisha reached 98.9%, 98.5%, 89.7%, and 84%, respectively, while those of Paotai, Mosuowan, Wulanwusu, Aksu, Kuche, Bachu, Makit, Yutian, and Aral were less than 30% (Table S1). This indicated that the explanatory power of phenology on yield differed greatly across sites: phenology served as a core driver of yield variation at some sites, whereas yield at other sites was more strongly regulated by other environmental or management factors. The total contribution rates of Zepu, Yopurga, Xinhe, and Yinjisha differed remarkably from those of the other sites. This spatial heterogeneity might be partially influenced by the length of the time series at each site (Figure 10). For Figure 10, the residual normality test (Shapiro–Wilk test), homoscedasticity test (Pearson correlation), and serial autocorrelation test (Durbin–Watson test) were conducted, all of which conformed to the classical assumptions of simple linear regression, validating the reliability of the significant regression results and the non-existence of false significance risk. The relatively short observation periods (11–15 years) at Zepu, Yopurga, Xinhe, and Yinjisha might have limited the capture of interannual environmental variability, leading to an overrepresentation of phenological effects in explaining yield variance. Thus, these high contribution rates should be interpreted with caution, as they might not reflect the long-term relative importance of phenology compared to other environmental or management factors.

For the above reasons, four sites (Zepu, Yopurga, Xinhe, and Yinjisha) should be excluded from the high-altitude group when calculating the average phenological contribution rates within the group. Meanwhile, Turpan should also be excluded, since the contribution rate of its Bol reached 26.8%, which was 20 percentage points higher than that of the Bol stage in other sites of the low-altitude group. Therefore, the mean values of contribution rates (%) of Sow, Eme, Squ, Flo, Bol, Mat, Sow–Eme, Eme–Squ, Squ–Flo, Flo–Bol, Bol–Mat, and total contribution rate (%) for the low-altitude group (and high-altitude group, respectively) were −0.675 ± 3.825 (−0.600 ± 3.231), −1.442 ± 3.629 (−1.989 ± 2.601), −0.458 ± 3.878 (−0.056 ± 3.274), −0.442 ± 3.859 (−0.450 ± 3.237), 0.300 ± 3.883 (0.300 ± 3.274), −1.683 ± 3.535 (1.300 ±3.007), 1.500 ± 3.594 (−0.356 ± 3.245), 1.208 ± 3.691 (−0.100 ± 3.262), 2.542 ± 2.965 (−0.589 ± 3.221), 3.183 ± 2.208 (0.567 ± 3.212), 0.058 ± 3.883 (0.961 ± 3.126), and 40.40833 ± 12.513 (36.867 ± 7.989). From these results, the altitudinal differentiation characteristics of the contribution rates of cotton phenological phases to lint yield can be concluded as follows: First, the total contribution rates (%) showed slight differences between the two groups, with the low-altitude group having a slightly higher mean value (40.41 ± 12.51) than the high-altitude group (36.87 ± 7.99). However, the standard deviation of the total contribution rate in the low-altitude group was significantly higher than that in the high-altitude group, indicating that the explanatory power of phenology on yield varied more greatly among sites in the low-altitude region, while the phenological contribution was relatively stable across sites in the high-altitude region. The phenological phases with the highest contribution rates were Flo–Bol for the low-altitude group and Eme for the high-altitude group, respectively. Second, distinct altitudinal differences were observed in the contribution direction and intensity of key phenological phases. The Mat exhibited completely opposite contribution directions between the two groups: it showed a negative contribution in the low-altitude group (−1.68 ± 3.54) but a positive contribution in the high-altitude group (1.30 ± 3.01). This could be attributed to insufficient accumulated temperature in high-altitude areas, where extending the maturity stage facilitates dry matter accumulation; in contrast, an overly prolonged maturity stage in low-altitude areas tends to be affected by high-temperature-induced forced ripening. For the Flo–Bol, both groups presented positive contributions, yet the contribution intensity of the low-altitude group (3.18 ± 2.21) was much higher than that of the high-altitude group (0.57 ± 3.21). Additionally, the Squ–Flo showed a positive contribution in the low-altitude group (2.54 ± 2.96) versus a negative contribution in the high-altitude group (−0.59 ± 3.22). Third, the contribution of most individual phenological phases was weak, and the within-group variability exceeded the between-group variability. For most phenological phases (e.g., Sow, Eme, Squ), the absolute values of the average contribution rates were less than 2, suggesting a limited direct impact on yield. Moreover, the standard deviation of each phenological phase in both groups was considerably higher than the absolute value of the mean contribution rate, which indicated that the phenological contribution had strong site-specificity within each group, and the differences among sites in the same altitude group were more significant than the differences between the two altitude groups. Finally, the contribution of phenological intervals was generally superior to that of individual phenological phases. In the low-altitude group, three key phenological intervals (Squ–Flo, Flo–Bol, Sow–Eme) all showed positive contributions, and their mean contribution rates were higher than those of most individual phenological phases. Even in the high-altitude group, where the overall contribution of phenological intervals was weaker, their performance was still better than that of the corresponding individual phenological phases, confirming that the duration of phenological stages had a more pronounced impact on yield than the occurrence time of single phenological events.

Key phenological phases for yield

Late-season phenological phases and their durations were consistently identified as the most impactful factors across sites. Specifically, the Bol–Mat and Flo–Bol contributed most positively to yield, highlighting the critical role of late-season dry matter accumulation and boll development in cotton lint yield.

Site-specific phenological effects

The Turpan site displayed a distinct contribution pattern, with only the Bol and Mat phases showing significant positive contributions to yield. This reflected the dominance of extreme arid conditions in Turpan, where late-season phenology directly determined yield, while the effects of early-season phenological phases were masked by the harsh local environment.

Direction of phenological contributions

Positive and negative values of the contribution rates indicated a consistent or opposite direction to the yield increase trend. The total contribution rate at each site was calculated as the sum of the absolute values of individual phenological contributions, ensuring that both positive and negative regulatory effects were accounted for in the overall explanatory power of phenology.

### 3.5. Relationship Between Altitude and Typical Yield Indicators

**Figure 11:**
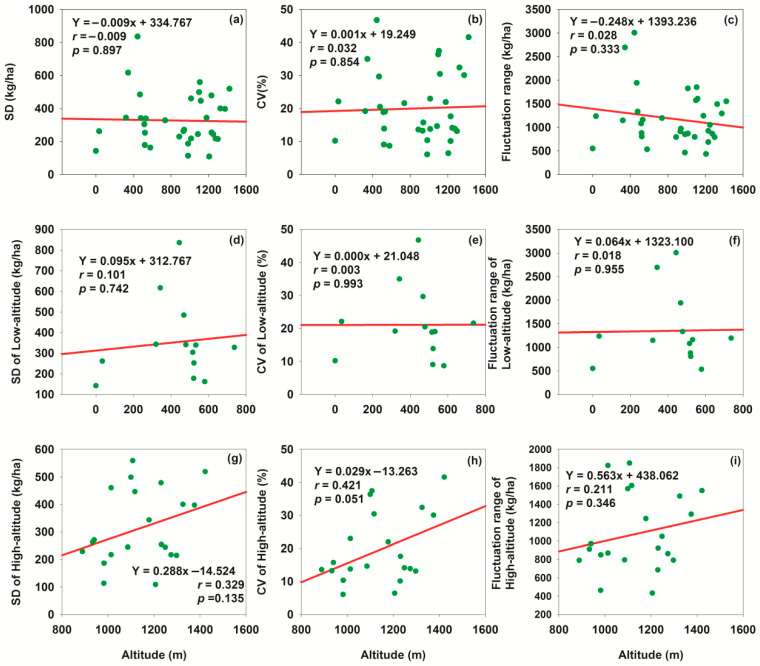
Relationships between altitude and interannual stability indicators of yield. (**a**) Relationship between altitude and SD of cotton lint yield at each station; (**b**) relationship between altitude and CV of cotton lint yield at each station; (**c**) relationship between altitude and fluctuation range of cotton lint yield at each station; (**d**) relationship between low altitude and SD of cotton lint yield at corresponding stations; (**e**) relationship between low altitude and CV of cotton lint yield at corresponding stations; (**f**) relationship between low altitude and fluctuation range of cotton lint yield at corresponding stations; (**g**) relationship between high altitude and SD of cotton lint yield at corresponding stations; (**h**) relationship between high altitude and CV of cotton lint yield at corresponding stations; (**i**) relationship between high altitude and fluctuation range of cotton lint yield at corresponding stations.

We assessed the interannual stability of yield along an elevation gradient using three quantitative metrics: standard deviation (SD), coefficient of variation (CV), and fluctuation range. For total yield (Figure 11a–c), no significant correlations were observed between altitude and SD (*r* = −0.009, *p* = 0.897), CV (*r* = 0.001, *p* = 0.854), or fluctuation range (*r* = 0.028, *p* = 0.333), demonstrating that the temporal stability of total yield remained consistent across the studied elevation gradient. Similarly, for low-altitude yield (Figure 11d–f), SD (*r* = 0.101, *p* = 0.742), CV (*r* = 0.003, *p* = 0.993), and fluctuation range (*r* = 0.018, *p* = 0.955) showed no significant trends with increasing elevation. In contrast, high-altitude biomass (Figure 11g–i) exhibited positive associations between these stability metrics and altitude, with SD (*r* = 0.329, *p* = 0.135), CV (*r* = 0.421, *p* = 0.051), and fluctuation range (*r* = 0.211, *p* = 0.346) all rising with increasing elevation. Notably, the correlation between CV and altitude approached statistical significance (*p* = 0.051), implying that interannual variability in high-altitude biomass tended to increase—conversely, stability tended to decrease—with ascending elevation. Collectively, these results indicated that the interannual stability of total and low-altitude yield was insensitive to altitude variation, whereas the stability of high-altitude biomass exhibited a decreasing trend with increasing elevation.

To investigate the effect of altitude gradient on cotton yield stability, inter-group difference analysis was performed for three core stability indicators (Standard Deviation, SD; Coefficient of Variation, CV; Annual Fluctuation Range) between low- and high-altitude groups (low altitude: *n* = 13; high altitude: *n* = 22, with unequal sample sizes). The testing procedure followed the sequence: Shapiro–Wilk normality test → Levene’s test for homogeneity of variances → independent samples *t*-test (Welch’s correction for heterogeneous variances). Bonferroni correction (k = 3, α_corrected ≈ 0.0167) was employed to control multiple testing errors, and the results are presented in Table 4.

As shown in Table 4, the means of all three stability indicators were higher in the low altitude group than in the high-altitude group. Intra-group variability was more pronounced in the low-altitude group for SD and Annual Fluctuation Range, while CV exhibited similar variability between the two groups. Nevertheless, no statistically significant inter-group differences were observed for any indicator after Bonferroni correction (all corrected *p*-values > 0.0167), with effect sizes indicating weak actual differences (Cohen’s d = 0.34~0.45).

In conclusion, the altitude gradient had no significant impact on cotton yield SD, CV, or Annual Fluctuation Range. Combined with previous correlation analysis between altitude and yield stability, this finding further confirmed that altitude exerted a limited regulatory effect on cotton yield stability.

## 4. Discussion

Numerous studies have focused on the impacts of climate change on cotton phenology [18,19,20,21]. All these findings focused on the extent to which climate change affects phenology, while failing to quantify the impact of phenological changes on cotton yield. The results of this study indicated that the Sow, Eme, Squ, and Flo dates showed an advancing trend, while the Bol and Mat dates exhibited a delaying trend. In terms of growth phases, the durations of Sow–Eme, Squ–Flo, Bol–Mat, and VGP presented a shortening trend, whereas those of Eme–Squ, Flo–Bol, RGP, and WGP showed an extending trend. Meanwhile, the cotton yield demonstrated an increasing trend. In addition, we also quantified the extent to which each one-day delay or extension of a phenological stage affects the increase or decrease in yield, as well as the contribution rate of each phenological stage to yield. These results can provide a decision-making basis for farmers and agricultural managers to adjust their farming practices.

The Flo–Bol stage serves as the critical period that dominates cotton yield development [3]. The contribution rate of the Flo–Bol stage in the low-altitude group of this study supported this view; however, in the high-altitude group, the Eme date exhibited the highest contribution rate, which does not support this view (Table S2). This striking altitudinal heterogeneity in yield-contributing phenological stages is primarily attributed to the divergent climate gradients shaped by altitude. Specifically, low-altitude regions are characterized by abundant thermal resources but frequent exposure to high-temperature stress (≥35 °C) during the Flo–Bol period [24]. Although heat stress could reduce pollen viability and boll-setting rate, the sufficient accumulated temperature in low-altitude areas still ensured that the Flo–Bol stage—the core period for boll formation and expansion—maintains its dominant role in yield accumulation. In contrast, high-altitude regions typically had lower temperatures and a longer growing season, where the Eme stage (emergence stage) determined the initial stand establishment and seedling vigor, which were prerequisites for subsequent growth and development. The relatively cool temperatures in high-altitude areas alleviated heat stress during the Flo–Bol stage but limited the overall growth duration; thus, a robust seedling establishment at the Eme stage became the bottleneck for yield formation, leading to its elevated contribution rate. This finding is consistent with the conclusion that altitude-driven climate factors (e.g., temperature lapse rate, solar radiation) regulate the spatial heterogeneity of cotton phenology [25]. Additionally, the differential responses may also be related to the adaptive traits of cotton varieties in different altitude zones: low-altitude cultivars may be bred for enhanced boll development potential, while high-altitude cultivars prioritize seedling cold tolerance to cope with the cool early-growing season. Collectively, these results highlight that the identification of yield-dominant phenological stages cannot be generalized across regions, and altitude-specific management strategies should be formulated accordingly.

Altitude, as a key driver of spatial heterogeneity in phenology, has attracted attention for its impacts on cotton production. Wang et al. [8] pointed out that in regions above 800 m altitude, where the average temperature in July was below 23 °C and the frost-free period was less than 150 days, cotton cultivation was not suitable. However, regions with an altitude above 762 m clearly exhibit the typical mountain climate characterized by high daytime temperatures and mild nighttime temperatures; this environment is more favorable to heat-sensitive varieties (e.g., certain Pima lines, 126–1, etc.), which show better yield performance and adaptability at high altitudes than at low altitudes, even though the low-temperature conditions in high-altitude areas will prolong the crop’s growing period [26]. However, in this study, all 22 experimental sites were at an altitude above 866.817 m, and cotton successfully completed its entire growing cycle at all these sites. The whole growing periods of the low-altitude group and the high-altitude group were 169.945 ± 16.435 days and 190.478 ± 7.584 days, respectively, with corresponding lint yields of 1716.936 ± 151.6014 kg ha^−1^ and 1599.557 ± 220.935 kg ha^−1^. Specifically, the whole growing period of the high-altitude group was indeed longer than that of the low-altitude group, while the lint yield of the high-altitude group was lower than that of the low-altitude group. Increased altitude leads to a reduction in air temperature, which in turn decreases accumulated temperature and ultimately prolongs the growing period of cotton; meanwhile, low nighttime temperatures in high-altitude areas reduce respiratory consumption and promote dry matter accumulation [26,27,28]. Altitude can affect the phenology and yield of cotton, but this environmental constraint can be addressed by breeding improved and adaptive varieties—for instance, early-maturing varieties are suitable for cultivation in high-altitude areas, while late-maturing varieties are more appropriate for low-altitude areas.

Although the annual shift is minimal, over the 43-year study period (1981–2023), the cumulative phenological change reaches approximately 0.86–1.72 days per 100 m altitude. For cotton—a crop highly sensitive to phenological synchronization with climate conditions—even such subtle cumulative shifts can alter the alignment between key growth stages (e.g., flowering-boll setting) and optimal environmental windows (e.g., thermal resource availability, precipitation periods). This misalignment, though incremental, may exacerbate the risk of exposure to extreme climates (e.g., high-temperature stress during boll development in low-altitude areas, low-temperature damage during the seedling stage in high-altitude areas) over time, which has practical implications for long-term cotton production adaptation.

The small effect size is inherently consistent with the regional characteristics of Xinjiang’s arid oases. Xinjiang’s cotton-producing areas are dominated by stable oasis ecosystems, where microclimate conditions (e.g., irrigation regulation, plastic film mulching) partially buffer the impact of altitude on temperature and phenology. The statistically significant yet small effects precisely reflect this buffering mechanism, providing critical insights for optimizing agronomic practices: for example, in high-altitude areas, slight adjustments to sowing dates (based on the quantified phenological shift rate) can compensate for the cumulative delay, ensuring synchronization with optimal growth conditions. This highlights that the small effect size itself carries practical guidance value for refined management.

Similar studies on cotton phenology in arid regions (e.g., Sun et al., [29]) have also reported small but significant phenological shifts driven by altitude, emphasizing that such subtle changes are typical in stable oasis agroecosystems. Our study further quantifies these shifts with long-term data, filling the gap in regional-specific quantitative parameters—these data can serve as a baseline for climate change adaptation models, supporting the formulation of long-term, targeted cultivation strategies rather than short-term agronomic adjustments.

The reported −0.25 kg ha^−1^ per 100 m is a per-altitude-unit change; when scaled to the actual altitude gradient of the study area (444.2 m, from the lowest to highest station), the cumulative yield change reaches −1.11 kg ha^−1^. For Xinjiang’s cotton-producing oases, where large-scale mechanized cultivation is prevalent (average field size > 50 ha), this translates to a cumulative yield difference of ~55.5 kg per field across the altitude gradient. While this is small relative to inter-annual yield variability driven by climate extremes (e.g., drought, high temperature), it is biologically and agronomically meaningful in the context of stable, long-term altitude-driven effects. The typical inter-annual yield variability in our study area is 5–8% (based on local agricultural statistics, 1981–2023), while the altitude-driven yield change accounts for ~1.2% of the average yield (4680 kg·ha^−1^). While this proportion is small, it represents a consistent, predictable driver of yield variation—distinct from stochastic climate variability. This predictability is practically valuable for long-term land use planning and breed selection: breeders can target varieties with enhanced tolerance to altitude-induced microclimate changes, and policymakers can incorporate this quantified rate into regional cotton production forecasting models.

The cotton phenological progression displays stage-dependent variations, with considerable spatial heterogeneity among the 35 monitoring sites. Such stage-specific differences may be attributed to the asymmetric responses of cotton growth stages to climate warming: earlier phenological phases (e.g., sowing, emergence) are more sensitive to the increase in spring temperature, which accelerates seed germination and seedling establishment, thus advancing these stages; in contrast, the delay in maturity is likely related to the prolonged reproductive growth period (as reflected in the lengthening of RGP), as higher temperatures during the late growing season may extend the boll-filling process [20,21,24,30]. In the future, cotton phenology can be regulated by cultivation practices such as adjusting sowing dates and breeding cultivars with longer growth periods [30]. Meanwhile, the substantial spatial variability across sites could be explained by the combined effects of altitude gradients and local agricultural management, for example, lower-altitude sites with better irrigation conditions may experience more significant advances in sowing dates due to earlier soil thawing, while higher-altitude sites (with cooler temperatures) tend to show delayed maturity to accumulate sufficient heat for boll opening [31].

The spatial heterogeneity of cotton phenological responses to climate change has become a core focus of regional cotton production adaptation research, with altitude identified as a key driving factor. Li et al. [25] argued that climate change exerts significantly heterogeneous impacts on cotton production: in low-altitude regions with sufficient heat, rising temperatures will advance cotton phenological phases and shorten the reproductive growth period, thereby leading to yield reduction; in contrast, in high-altitude regions, moderate warming can alleviate low-temperature stress during the early growth stage of cotton, which is conducive to biomass accumulation. This differentiation was essentially attributed to the combined effects of altitude-dependent climate factors, including temperature lapse rate, solar radiation intensity, and soil moisture conditions [25,32]. Specifically, high-altitude areas typically had stronger solar radiation but lower ambient temperatures, where the low-temperature constraint often limited boll development and delayed maturity; while low-altitude areas were characterized by abundant thermal resources but faced the risk of high-temperature stress, which accelerated the transition of phenological stages and compressed the reproductive growth period critical for yield formation [25]. These findings collectively confirmed that altitude-driven climate gradients were the fundamental cause of the spatial heterogeneity of cotton phenology, highlighting the necessity of formulating differentiated cultivation strategies based on altitude characteristics to cope with climate change impacts [25,32]. For instance, in high-altitude cotton-growing regions, agronomic practices such as selecting late-maturing varieties tolerant to low temperatures and adopting plastic film mulching to improve soil temperature could be prioritized; whereas in low-altitude regions, optimizing irrigation schedules and breeding heat-resistant cultivars would be more effective in mitigating yield losses caused by high-temperature stress. Moreover, these site-specific strategies should be integrated with climate change projections to enhance the long-term adaptability and sustainability of cotton production systems across different altitude gradients. We noted that our findings were consistent with Sun et al. [29], who reported that elevation-induced thermal variations regulate crop yield by modifying key growth stages. We emphasized that clarifying this elevation–phenology–yield chain was critical for regional cotton management—for high-altitude areas, the yield loss caused by shortened reproductive growth period can be mitigated by selecting early-maturing varieties; for low-altitude areas, targeted regulation of phenological stages (e.g., optimizing sowing dates) could reduce the negative impact of spatial heterogeneity on yield stability.

Although altitude exerted a regulatory effect on the correlation with cotton yield variability, this effect did not reach a statistically significant level, and the above correlation tended to be stronger in high-altitude areas than in low-altitude areas. Asse et al. [10] argued that warmer springs advance tree spring phenology (e.g., leaf expansion), while warmer winters offset this effect; it also confirms that global warming weakens spring phenology’s altitudinal gradient, helping clarify tree phenology’s response to climate change in high-altitude areas and supplementing relevant literature. Qiu et al. [11] quantified the distribution patterns of crop phenology (cropping systems) in different altitudinal regions of China and provided high-precision remote sensing monitoring evidence. Zheng et al. [12] revealed the regulatory pathway of altitude on crop flowering phenology at the molecular level. Low-temperature stress caused by increased altitude selects turnip individuals carrying functional FRIa variants—such genetic variations can adjust crop flowering time by regulating the expression of flowering repressor genes.

Our study identified both robust spatial patterns and weak marginal effects when examining the relationships among altitude, cotton phenology, and yield in arid oases. The robust results include the statistically significant associations between altitude and cotton phenology, as well as the clear spatial heterogeneity of altitude’s regulatory effects across observation sites. These consistent patterns provide new evidence for the importance of altitude as a key environmental factor influencing cotton growth in arid regions. In contrast, the magnitudes of phenological and yield changes associated with altitude were relatively small, representing weak but statistically significant effects. Specifically, phenology shifted by only 0.02–0.04 days year^−1^ per 100 m elevation, and yield changed by −0.25 kg ha^−1^ per 100 m. Although these per-unit effects appear modest in absolute terms, they reflect stable, long-term environmental constraints imposed by altitude, rather than short-term climatic fluctuations. By clearly distinguishing robust spatial patterns from weak marginal effects, this study improves the interpretation of altitude-driven influences on cotton growth. Even though the per-unit effect sizes are small, the consistent spatial structure of these relationships carries meaningful scientific implications for understanding regional differences in crop phenology and for guiding site-specific cotton management in arid oasis systems.

The interactive relationships among altitude, phenology, and cotton yield in arid oases—core to this study—are well supported by research across diverse crop systems and regions. Numerous studies have confirmed that altitude, a key topographical factor, regulates crop growth, phenology, and yield primarily by modifying thermal conditions, with temperature and growing degree days (GDD) as core intermediate drivers. Cleland et al. [2] comprehensively reviewed plant phenological responses to global change, explicitly noting that temperature and GDD are critical climatic drivers of phenology, and that altitude influences phenology mainly by altering temperature and GDD regimes. This underpins the core theoretical framework of our study: “altitude → temperature/GDD → cotton phenology → cotton yield”.

Research on food crops and fruit trees has further verified the universality of the altitude–temperature–GDD relationship, informing inferences about altitude’s regulatory mechanisms on cotton in arid oases. Wang et al. [5] confirmed that altitude affects spring wheat growth via temperature differences; though GDD was not explicitly addressed, the temperature-driven mechanism aligns with our study’s core logic. Wei et al. [6] found that increasing altitude significantly reduces temperature, alleviating loquat heat damage, and their analysis of temperature and accumulated temperature (GDD) along altitudinal gradients closely aligns with our focus on altitude–temperature–GDD linkages in arid oases. Wang et al. [7,8] further identified altitude, temperature, and GDD as key drivers of crop phenology and planting suitability in maize and cotton studies. Notably, Wang et al. [8] used altitude, temperature, and ≥10 °C accumulated temperature (GDD) for fine-scale climate zoning of cotton planting in Shawan, Xinjiang, demonstrating that altitude shapes regional thermal conditions (temperature and GDD) to determine suitable cotton-growing areas—directly supporting the practical relevance of our study on cotton in arid Xinjiang oases.

Sudjatmiko and Chozin [9] provided direct empirical evidence for the altitude–temperature–GDD relationship in sweet corn, quantitatively showing that higher altitude reduces air temperature and, consequently, GDD accumulation rate and total across growth stages. This informs our quantification of interactive relationships among altitude, GDD, cotton phenology, and yield. Additional support for altitude–temperature linkages comes from tree phenology and plateau-adaptive plant studies: Asse et al. [10] showed that altitude mediates temperature regimes and regulates tree phenological responses to climate change, while Zheng et al. [12] found that higher altitude (and lower temperature) delays turnip flowering on the Qinghai-Xizang Plateau. Though neither study analyzed GDD, their confirmation of altitude–temperature linkages reinforces the rationale for using temperature and GDD as intermediate variables in our research. Even early cotton research [27] demonstrated that altitude-induced temperature differences determine cotton sowing dates, seedling establishment, and phenological progress—consistent with our conclusion that altitude affects cotton phenology and yield via thermal regulation, reflecting long-standing academic attention to altitude’s impact on cotton production.

Studies on grapes, another economic crop, have supplemented the physiological mechanisms underlying the altitude–temperature–GDD relationship, providing valuable insights for understanding cotton responses in arid oases. Arrizabalaga-Arriazu et al. [33,34] simulated climate change scenarios and found that GDD accumulation (typical of low-altitude, high-temperature environments), alone or combined with elevated CO_2_, alters grape growth, carbon allocation, and fruit quality. These findings indirectly support the altitude–temperature–GDD nexus: higher altitude reduces temperature and GDD accumulation, mitigating high-temperature adverse effects on crop growth and quality—providing a core physiological reference for our exploration of how altitude regulates cotton yield via temperature/GDD and phenology in arid oases. Most importantly, Arias et al. [35] specifically addressed altitude–temperature–GDD relationships in their review, clarifying that higher altitude reduces ambient temperature and GDD accumulation, delaying crop ripening and maintaining product quality. This strongly supports our study’s core logic and provides a theoretical basis for exploring altitude-based adaptation strategies for cotton in arid oases under climate change.

In summary, existing research across crops (grains, fruits, and economic crops) and regions has fully confirmed close linkages among altitude, temperature, GDD, and crop phenology, with some studies directly focusing on cotton and arid regions—laying a solid theoretical and empirical foundation for our work. Building on this, our study focuses on arid oases, quantifies interactive relationships among altitude, phenology, and cotton yield, and clarifies the specific path by which altitude regulates cotton yield via temperature/GDD and phenology. This enriches research on altitude effects on economic crops in arid regions and provides practical guidance for rational cotton planting layout and yield improvement in arid oases.

Three studies—Yang et al. [36], Wang et al. [37], and Ganjurjav et al. [38]—collectively confirm the altitude–phenology–yield correlation from diverse regions, vegetation types, and perspectives, providing multi-dimensional support for this study’s core conclusions. Yang et al. [36] clarified the altitude–temperature/moisture–phenology–yield pathway based on long-term (1976–2006) alpine meadow observations, addressing short-term study limitations and aligning with this research’s logic. Wang et al. [37] extended this scope to cash crops via long-term (2005–2010) cotton observations across two altitude gradients (1305 m, 1315 m), demonstrating altitude’s direct impact on cotton yield and confirming the universal regulatory role of altitude across vegetation types. Ganjurjav et al. [38] further supplemented the moderating role of climate warming in this relationship, revealing its complexity and environmental dependence, while verifying the altitude-driven mechanism proposed by Yang et al. [36].

Collectively, these studies form a mutually supportive evidence chain: Yang et al. [36] established the core logic, Wang et al. [37] provided cash crop-specific evidence, and Ganjurjav et al. [38] added climate warming insights. All reflect the key pathway—altitude influences phenology via environmental factors, then yield, consistent with this study. However, they highlight common limitations: most focus on single vegetation types or regions, with insufficient quantitative research. This points to future directions: quantifying altitude gradient effects and considering multiple environmental factors. At present, long-term positioning studies on the altitude–phenology–yield relationship are still relatively limited, and the conclusions of this study need to be further verified by combining more long-term observation data in the future.

## 5. Conclusions

Interannual variation of phenological phases and yield trend: In the oasis cotton fields of the study area, the Sow, Eme, Squ, and Flo dates slightly advanced, while the Bol and Mat dates were slightly delayed. The duration of the Flo–Bol, RGP, and WGP was extended significantly, whereas the period of Squ-Eme, Squ–Flo, and the VGP was shortened slightly. The lint yield showed a significant increasing trend over the years (24.061 kg ha^−1^ year^−1^).

Differential impacts of phenological characteristics on yield: Delays in the phenological phases from Sow to Bol all led to yield reductions, with the delay in Eme exerting the most prominent negative impact (−9.780 kg ha^−1^ per day). In contrast, a delay in Bol resulted in a slight yield increase. Extensions in the Eme–Squ, Bol–Mat, RGP, and WGP promoted yield increases, while extensions in Sow–Eme, Squ–Flo, and VGP suppressed yield.

Systematic impact of altitude: For every 100 m increase in altitude, all phenological phases were slightly delayed, the durations of Eme–Squ, Flo–Bol, Bol–Mat, and RGP were slightly extended, but the durations of Sow–Eme, Squ–Flo, and WGP were shortened. The lint yield decreased slightly (0.250 kg ha^−1^ per 100 m). The spatial heterogeneity of phenology and yield was higher in low-altitude areas, providing support for zonal management.

Combined with the limitations of this study and the current research status, the following future research directions are proposed. First, expand the research area and sample size to further cover elevational gradients under different climate zones and soil types, verify the universality of the conclusions of this study, and reduce the deviation caused by regional limitations. Second, further explore the internal physiological and biochemical mechanisms by which altitude affects cotton phenology and yield, and clarify the specific pathways by which altitude affects cotton phenology and yield by regulating key physiological processes such as cotton photosynthetic efficiency, nutrient absorption, and stress resistance, combined with molecular biology, physiological ecology, and other technical means. Third, combined with climate change scenarios, carry out long-term positioning research on the interactive effects of altitude and climate change on cotton phenology and yield, predict the changes of cotton phenology and yield in different altitude regions under the background of future global warming, and provide scientific support for the long-term adaptive regulation of cotton cultivation. Fourth, strengthen the research on multi-factor coupling, comprehensively consider the interactive effects of altitude with cultivation measures such as planting density, fertilization level, and irrigation method, and construct a comprehensive, high-quality, and high-yield cotton cultivation technology system based on elevational gradients. Fifth, supplement the research on the impact of altitude on cotton quality (such as fiber length, breaking strength, etc.), improve the comprehensive evaluation system of cotton growth and output under elevational gradients, and provide a more comprehensive theoretical basis for improving the quality and efficiency of cotton cultivation.

## Figures and Tables

**Table 1:** The cotton station information in Xinjiang, China.

Station Code	Station Name	Latitude (°)	Longitude (°)	Altitude (m)	Time Series of Yield and Phenology (Year)
51238	Bole	44.9	82.07	532.2	1991–2023
51248	Jinghe	44.62	82.9	320.1	1991–2023
51346	Wusu	44.43	84.67	478.7	1991–2023
51352	Paotai	44.63	85.18	342.8	1981–2023
51356	Mosowan	44.32	86.05	443.7	1983–2023
51357	Shawan	44.20	85.37	522.2	1997–2023
51358	Wulanwusu	44.28	85.82	468.5	1981–2023
51359	Manas	44.32	86.2	520.3	2000–2023
51367	Hutubi	44.02	87.43	579.3	2005–2023
51368	Changji	44.07	87.32	515.7	1999–2023
51568	Heshuo	42.25	86.8	1085.4	1997–2023
51571	Tuokexun	42.48	88.38	0.1	1997–2023
51573	Turpan	42.93	89.2	34.5	1981–2023
51628	Aksu	41.12	80.38	1107.1	1981–2023
51633	Wensu	41.78	81.9	1230	2000–2023
51636	Xinhe	41.25	82.38	981.3	2009–2023
51639	Shaya	41.23	82.77	982.8	1997–2023
51642	Luntai	41.82	84.27	982	1981–2023
51644	Kuche	41.7167	82.9667	1099.2	1982–2023
51655	Yuli	41.37	86.22	932.7	1997–2023
51656	Korla	41.73	85.82	939.7	1981–2023
51708	Akto	39.15	75.95	1325.1	1986–2023
51716	Bachu	39.8	78.57	1116.5	1981–2023
51720	Yopurga	39.217	76.817	1206	2013–2023
51730	Aral	40.5	81.05	1013	1983–2023
51733	Awat	40.5	81.05	1013	2000–2023
51777	Ruoqiang	39.02	88.18	888.2	1983–2023
51804	Yingjisha	38.93	76.17	1297.5	2009–2023
51810	Makit	38.92	77.63	1178.2	1984–2023
51811	Shache	38.43	77.27	1231.2	1993–2023
51818	Zepu	38.2	77.26	1273.7	2011–2023
51828	Hetian	37.13	79.93	1375	1986–2023
51855	Qiemo	38.09	85.55	1248.4	1981–2023
51931	Yutian	36.85	81.65	1422	1982–2023
52203	Hami	42.49	93.31	737.2	1985–2023

**Table 2:** Statistics of cotton phenological variations and stage durations, and average trends in changes of cotton phenology across 35 sites in arid oases zones (days year^−1^).

Phenology	Insignificant Increase	Significant Increase	Insignificant Decrease	Significant Decrease	Mean ± SD	Max.~Min.
Sowing	11	3	8	13	0.148 ± 0.185	0.900~−0.574
Emergence	8	3	13	11	0.121 ± 0.166	0.868~−0.559
Squaring	11	2	14	8	0.134 ± 0.168	0.128~−0.635
Flowering	11	2	15	7	0.078 ± 0.128	0.452~−0.483
Boll opening	11	5	12	7	0.242 ± 0.205	1.132~−0.968
Maturity	13	4	10	8	0.194 ± 0.179	0.907~−1.336
Sow–Eme	12	6	11	6	0.085 ± 0.087	0.500~−0.318
Eme–Squ	15	5	10	5	0.162 ± 0.150	0.775~−0.929
Squ–Flo	13	3	10	9	0.089 ± 0.118	0.307~−0.951
Flo–Bol	15	8	8	4	0.209 ± 0.125	1.009~−0.779
Bol–Mat	11	7	12	5	0.284 ± 0.299	0.921~−2.373
VGP	7	9	12	7	0.176 ± 0.180	0.726~−0.873
RGP	14	7	13	1	0.218 ± 0.158	0.823~−1.364
WGP	12	8	13	1	0.264 ± 0.229	0.716~−2.236

Mean ± SD means Mean trend ± SD; “+”represents phenology advancement or prolongation, and “−” represents phenology delay or shortening. “Max.” and “Min.” represent maximum and minimum trend values, respectively.

**Table 3:** Statistics on the number of sites affected by a 1-day delay or lengthening in cotton phenology on yield, and impacts of a 1-day phenological delay or growth period lengthening on cotton lint yield (kg ha^−1^ day^−1^).

Phenology	Insignificant Increase	Significant Increase	Insignificant Decrease	Significant Decrease	Mean ± SD	Max.~Min.
Sowing	10	2	12	11	4.653 ± 12.273	20.985~−47.660
Emergence	7	2	18	8	1.965 ± 11.745	14.042~−40.703
Squaring	15	1	9	10	6.278 ± 11.780	24.178~−43.677
Flowering	10	2	15	8	3.391 ± 10.832	16.295~−43.233
Boll opening	13	4	15	3	5.105 ± 6.000	22.868~−32.225
Maturity	9	5	19	2	6.312 ± 5.524	24.861~−18.377
Sow–Eme	16	1	14	4	10.139 ± 15.863	46.604~−78.815
Eme–Squ	14	5	12	4	8.714 ± 7.971	37.508~−37.854
Squ–Flo	16	1	15	3	9.598 ± 14.020	71.833~−64.099
Flo–Bol	15	9	9	2	10.348 ± 4.579	51.528~−24.262
Bol–Mat	12	8	12	3	6.537 ± 4.527	31.721~−16.055
VGP	13	4	14	4	7.464 ± 10.531	22.480~−78.700
RGP	16	5	12	2	7.179 ± 3.787	34.170~−17.080
WGP	15	6	14	0	6.223 ± 3.058	25.211~−11.100

Note: Positive values indicate an increase in lint yield, while negative values indicate a decrease caused by a 1-day delay or lengthening in phenological stages. Mean ± SD means Mean yield increase ± SD; “+” represents increased lint yield, and “−” represents decreased lint yield. “Max.” denotes the maximum value of yield increase, and “Min.” denotes the maximum absolute value of yield decrease.

**Table 4:** Relevant indicators of inter-group differences between low- and high-altitude groups.

Analysis Items	SD (kg ha^−1^)	CV (%)	Annual Fluctuation Range (kg ha^−1^)
Mean (Low/High Altitude)	368.54/316.81	21.23/16.67	1389.35/1144.78
Inter-group Mean Difference	51.73 kg ha^−1^	4.56	244.57 kg ha^−1^
Intra-group SD (Low/High Altitude)	187.92/136.57	10.58/10.42	721.58/415.62
Variance Homogeneity (Levene’s Test)	Homogeneous (*F* = 1.58, *p* = 0.21)	Homogeneous (*F* = 0.004, *p* = 0.95)	Heterogeneous (*F* = 4.82, *p* = 0.035)
Testing Method	Independent Samples *t*-test	Independent Samples *t*-test	Welch’s Corrected *t*-test
t-value/df	1.01/33	1.32/33	1.27/18.64
Raw *p*-value	0.32	0.20	0.22
Corrected *p*-value (k = 3)	0.96 > 0.0167	0.60 > 0.0167	0.66 > 0.0167
Effect Size (Cohen’s d)	0.34 (Small Effect)	0.45 (Small-to-Medium Effect)	0.43 (Small-to-Medium Effect)

Note: For homogeneous variances (SD and CV), degrees of freedom (df) = total sample size − 2 = 35 − 2 = 33; for heterogeneous variances (Annual Fluctuation Range), df = 18.64 was calculated via Welch’s correction.

## Data Availability

Supplementary datasets have been deposited in ScienceDB under the accession number DOI: 10.57760/sciencedb.35686.

## Supplementary Materials

The following supporting information can be downloaded at: https://www.mdpi.com/article/10.3390/plants15050824/s1, Table S1: Phenology mean dates. Table S2: Contribution rates of phenology.
