# Supplementary material for: Altitude, Phenology, and Cotton Yield in Arid Oases: Quantifying Their Interactive Relationships
Source: Plants (Basel). 2026 Mar 7;15(5):824. doi: 10.3390/plants15050824 (PMC12986762; doi:10.3390/plants15050824)
Supplement: Supplementary file 1 [file plants-15-00824-s001.zip › Table S1. phenology mean dates.pdf]

Station	Sowing	Emergence	Squaring	Flowering	Boll splitting	Maturity
Turpan	April 8th	April 21st	May 30th	June 20th	August 7th	October 31st
Tuokexun	April 18th	April 29th	June 9th	June 26th	August 11th	October 20th
Hami	April 20th	May 4th	June 14th	July 4th	September 5th	October 12th
Changji	April 23rd	May 5th	June 21st	July 9th	September 8th	October 15th
Hutubi	April 18th	May 2nd	June 21st	July 8th	September 6th	October 11th
Manas	April 19th	May 1st	June 12th	July 4th	September 1st	October 8th
Bole	April 23rd	May 6th	June 17th	July 11th	September 9th	October 13th
Jinghe	April 24th	May 6th	June 17th	July 11th	September 8th	October 14th
Korla	April 18th	April 29th	June 8th	July 5th	September 5th	October 18th
Luntai	April 9th	April 24th	June 13th	July 7th	September 17th	October 25th
Yuli	April 18th	May 1st	June 12th	July 5th	September 9th	October 18th
Ruoqiang	April 20th	May 2nd	June 13th	July 5th	September 3rd	October 15th
Qiemo	April 19th	April 30th	June 12th	July 6th	September 12th	October 14th
Heshuo	April 18th	April 29th	June 10th	July 6th	September 9th	October 12th
Aksu	April 14th	April 26th	June 8th	July 7th	September 15th	October 25th
Kuche	April 12th	April 25th	June 8th	July 6th	September 11th	October 20th
Wensu	April 14th	April 28th	June 9th	July 7th	September 11th	October 26th
Shaya	April 9th	April 20th	June 3rd	July 3rd	September 11th	October 24th
Xinhe	April 20th	May 4th	June 15th	July 10th	September 7th	October 27th
Awat	April 13th	April 25th	May 28th	June 23rd	August 31st	October 23rd
Akto	April 11th	April 25th	June 8th	July 8th	September 11th	October 20th
Zepu	April 4th	April 17th	June 8th	July 5th	September 8th	October 27th
Shache	April 6th	April 17th	May 29th	June 29th	September 7th	October 21st
Bachu	April 5th	April 20th	May 31st	June 29th	September 1st	October 24th
Yinjisha	April 10th	April 19th	June 7th	July 4th	September 11th	October 28th
Makit	April 9th	April 21st	June 4th	June 29th	September 1st	October 19th
Yopurga	April 6th	April 19th	June 7th	July 2nd	September 3rd	October 27th
Hetian	April 4th	April 18th	June 4th	July 1st	September 11th	September 16th
Yutian	April 9th	April 20th	June 4th	July 3rd	September 13th	October 14th
Wusu	April 20th	May 1st	June 11th	July 6th	September 4th	October 11th
Shawan	April 19th	April 30th	June 8th	July 4th	September 2nd	October 14th
Wulanwusu	April 22th	May 6th	June 8th	July 7th	September 6th	October 9th
Mosuowan	April 16th	May 1st	June 8th	July 4th	September 1st	October 7th
Paotai	April 19th	May 4th	June 8th	July 5th	September 3rd	October 9th
Alar	April 16th	April 30th	June 3rd	July 2nd	September 10th	October 20th
