# Supplementary material for: Altitude, Phenology, and Cotton Yield in Arid Oases: Quantifying Their Interactive Relationships
Source: Plants (Basel). 2026 Mar 7;15(5):824. doi: 10.3390/plants15050824 (PMC12986762; doi:10.3390/plants15050824)
Supplement: Supplementary file 1 [file plants-15-00824-s001.zip › Table S2. Contribution rates of phenology.pdf]

	Sow	Eme	Squ	Flo	Bol	Mat	Sow-Eme	Eme-Squ
**Turpan**			-0.5		-26.8			
**Tuokexun**	-4.8	-5	-5	-4.8	-4.9	-5.1	-5	-4.8
**Hami**	-2.8	-2.8	2.8	2.8	2.8	-2.8	2.8	2.8
**Changji**	4.5	-4.5	-4.5	-4.5	4.5	4.5	-4.5	-4.5
**Hutubi**	5.9	5.9	-5.9	-5.9	-5.9	-5.9	5.9	-5.9
**Manas**	-5	-5	5	5	5	-5	4.9	5
**Bole**	-3.3	-3.3	-3.3	-3.3	-3.3	-3.3	3.3	3.3
**Jinghe**	-3.3	-3.3	-3.3	-3.3	-3.3	-3.3	3.3	3.3
**Wusu**	-3.3	-3.3	-3.3	-3.3	-3.3	-3.3	3.3	3.3
**Shawan**	-4	-4	4	4	4	-4	-4	4
**Wulanwus**	2.5	2.5	2.5	2.5	2.5	2.5	2.5	2.5
**Mosuwan**	2.6	2.6	2.6	2.6	2.6	2.6	2.6	2.6
**Paotai**	2.9	2.9	2.9	2.9	2.9	2.9	2.9	2.9
**Korla**	-3.7	-3.6	3.6	3.6	3.6	3.7	3.6	3.5
**Luntai**	-3.7	-3.7	3.6	-3.5	-3.7	3.6	-3.6	3.6
**Yuli**	-4.3	-4.3	4.3	4.3	4.4	4.3	-4.3	4.3
**Ruoqiang**	-3.4	-3.4	-3.4	-3.4	3.4	-3.4	3.3	-3.4
**Qiemo**	-3.3	-3.3	-3.3	-3.3	3.3	-3.3	-3.3	-3.3
**Heshuo**	-4	-4	4	4	4	4	4	4
**Akau**	-2.6	-2.6	-2.6	-2.6	-2.6	2.5	-2.5	-2.5
**Kuche**	2.6	-2.6	-2.6	-2.6	-2.6	2.6	-2.6	-2.6
**Wensu**	4.5	-4.5	4.5	4.5	-4.5	4.5	-4.5	4.5
**Shaya**	4	4	4	4	4	4	4	-4
**Akto**	2.8	2.8	2.8	2.8	2.8	2.8	-2.8	2.8
**Awat**	4.5	4.5	4.5	4.5	-4.5	-4.5	4.5	4.5
**Shache**	-3.7	-3.6	-3.7	-3.7	3.7	3.7	3.7	-3.7
**Bachu**	-2.4	-2.4	-2.4	-2.4	-2.4	2.4	-2.4	-2.4
**Makit**	2.6	2.6	-2.6	-2.6	2.6	2.6	-2.6	-2.6
**Hetian**	2.8	-2.8	-2.8	-2.8	2.8	2.8	2.8	-2.8
**Yutian**	2.7	-2.7	-2.7	-2.7	-2.7	-2.7	-2.7	-2.7
**Aral**	-2.6	-2.6	-2.6	-2.6	-2.6	-2.6	2.6	-2.6
**Xinhe**	8.3	8.2	-8.1	-8.1	8.2	8.2	8.2	-8.2
**Zepu**	9	9	9	9	9	9	-9	9
**Yopurga**	11	11	11	11	11	-10.9	-10.6	
**Yinjisha**	-7.7	-7.6	-7.7	-7.7	-7.6	-7.6	7.6	7.7

Squ-Flo	Flo-Bol	Bol-Mat	Total
	-3	-1	31.3
5	4.7	4.9	54
-2.8	2.8	-2.8	30.8
-4.5	4.5	-4.5	49.5
5.9	5.9	-5.9	64.9
5	5	-4.9	54.8
3.3	-3.3	3.3	36.3
3.3	3.3	3.3	36.3
3.3	3.3	3.3	36.3
4	4	-4	44
2.5	2.5	2.5	27.5
2.6	2.6	2.6	28.6
2.9	2.9	2.9	21.9
-3.6	3.6	-3.6	39.7
-3.6	-3.6	3.6	39.8
-4.3	4.2	-4.3	47.3
3.4	3.4	-3.4	37.3
3.3	3.2	-3.3	36.2
-4	4	4	44
2.6	-2.6	2.5	28.2
2.6	-2.6	2.6	28.6
4.5	-4.5	4.5	49.5
4	4	-4	48
-2.8	2.8	2.8	30.8
-4.5	-4.5	-4.5	49.5
3.7	3.7	3.7	40.6
2.4	2.4	2.4	26.4
-2.6	-2.6	2.6	28.6
-2.8	2.8	2.8	30.8
-2.7	2.7	2.7	29.7
-2.6	-2.6	2.6	28.6
7.9	8.2	8.1	89.7
-9	8.9	-9	98.9
-11		-11	98.5
-7.6	-7.6	7.6	84
